# Acid sphingomyelinase deficiency enhances myelin repair after acute and chronic demyelination

**DOI:** 10.1371/journal.pone.0178622

**Published:** 2017-06-05

**Authors:** Marwan Chami, Ramona Halmer, Laura Schnoeder, Katrin Anne Becker, Carola Meier, Klaus Fassbender, Erich Gulbins, Silke Walter

**Affiliations:** 1 Department of Neurology, Saarland University Hospital, Homburg, Germany; 2 Department of Molecular Biology, University Hospital Essen, University of Duisburg-Essen, Essen, Germany; 3 Institute of Anatomy and Cell Biology, Saarland University, Homburg, Germany; 4 Department of Surgery, University of Cincinnati, Cincinnati, Ohio, United States of America; 5 Department of Medicine, Royal Melbourne Hospital, University of Melbourne, Parkville, Victoria, Australia; 6 Florey Institute of Neuroscience and Mental Health, University of Melbourne, Parkville, Victoria, Australia; Hannover Medical School, GERMANY

## Abstract

The cuprizone animal model, also known as the toxic demyelination model, is a well-reproducible model of demyelination- and remyelination in mice, and has been useful in studying important aspect of human demyelinating diseases, including multiple sclerosis. In this study, we investigated the role of acid sphingomyelinase in demyelination and myelin repair by inducing acute and chronic demyelination with 5- or 12-week cuprizone treatment, followed by a 2-week cuprizone withdrawal phase to allow myelin repair. Sphingolipids, in particular ceramide and the enzyme acid sphingomyelinase, which generates ceramide from sphingomyelin, seem to be involved in astrocyte activation and neuronal damage in multiple sclerosis. We used immunohistochemistry to study glial reaction and oligodendrocyte distribution in acid sphingomyelinase deficient mice and wild-type C57BL/6J littermates at various time intervals after demyelination and remyelination. Axonal injury was quantified using amyloid precursor protein and synaptophysin, and gene expression and protein levels were measured using gene analysis and Western blotting, respectively. Our results show that mice lacking acid sphingomyelinase had a significant increase in myelin recovery and a significantly higher oligodendrocyte cell count after 2 weeks remyelination compared to wild-type littermates. Detrimental astroglial distribution was also significantly reduced in acid sphingomyelinase deficient animals. We obtained similar results in experiments using amitriptyline to inhibit acid sphingomyelinase. These findings suggest that acid sphingomyelinase plays a significant role in myelin repair, and its inhibition by amitriptyline may constitute a novel therapeutic approach for multiple sclerosis patients.

## Introduction

Demyelination is a key feature of multiple sclerosis (MS), an autoimmune disease that is the main cause of permanent disability in young adults. [1] Histopathologically, MS is associated with the widespread presence of demyelinated plaques, ultimately leading to oligodendrocyte loss and neurodegeneration. Depending on the disease stage, mechanisms that are associated with demyelination in MS include, among others, invasion of auto-aggressive leukocytes, toxic accumulation of oligodendrocyte-released iron, oxidative stress, mitochondrial in jury and microglial activation. [2] These mechanisms activate astrocytes, which appear to play both beneficial and detrimental roles in MS disease progression. [3, 4, 5, 6, 7] To date, there are no therapeutic options to control demyelination in MS, nor treatments to repair myelin damage sufficiently in chronic MS.

CNS demyelination can be investigated by treatment with cuprizone (*bis*-cyclohexanone oxaldihydrazone), a toxic copper-chelating agent. In animal models, cuprizone treatment, is a standard method for inducing demyelination as part of the chronic phase of multiple sclerosis, resulting in astrocyte activation [8] and strong demyelination with oligodendrocyte cell death in distinct brain areas, but not in spinal cord. Myelin recovery can occur after cuprizone withdrawal, making the cuprizone model useful for investigating both acute demyelination and myelin repair mechanisms.

Acid sphingomyelinase (Asm), a lysosomal enzyme, hydrolyses the membrane lipid sphingomyel into ceramide. [9, 10, 11, 12] A recent study demonstrated increased Asm activity in astrocytes in active MS lesions. [13] Other studies have shown ceramide to be important in the regulation and induction of oligodendrocyte cell death in human MS brains. [14]

Asm/ceramide may play a regulatory role in MS pathogenesis; investigation of their role may support the development of novel therapeutic strategies. To investigate the role of the Asm/ceramide system in demyelination, we examined the effects of genetic deficiency or pharmacological inhibition of Asm on the pathophysiological features of remyelination in the cuprizone-treatment animal model.

## Materials and methods

### Animals

All animal experiments were approved by the Ethical Review Committee of the Regional Council in Saarland, Germany. *In vivo* experiments were performed with 8-week-old male acid sphingomyelinase deficient mice (*Asm*^-/-^; protein: Asm, gene symbol: *Smpd1*) and wild-type (C57BL/6J) littermates obtained from our breeding facility. All mice were maintained in a pathogen-free environment; food and water were available ad *libitum*.

### Cuprizone-induced demyelination and amitriptyline treatment

Mice were fed with powdered food supplemented with 0.2% cuprizone (bis-cyclohexanone-oxaldihydrazone, Sigma-Aldrich, St. Louis, MO, USA) for 5 weeks (acute demyelination) or for 12 weeks (chronic demyelination). Afterwards, animals were fed with normal diet for the following 2 weeks to allow remyelination as previously shown. [8] Pharmacological Asm inhibition was achieved during the 2-week-recovery phase by twice daily intraperitoneal injections with 25 mg/kg amitriptyline dissolved in sterile distilled water, to insure the drug’s full function as previously described. [15] PBS injections were administered as control. The twice-daily dosing schedule was chosen to maintain a constant plasma level of amitriptyline as described earlier. [15] Animals subjected to a cuprizone-free diet served as untreated (naïve) controls; additional controls were animals given a cuprizone-free diet who received amitriptyline treatment. To evaluate amitriptyline’s effect in non-demyelinating conditions, amitriptyline-injected mice were compared to naïve controls.

### Immunohistochemistry

For immunohistochemical analysis, animals were sacrificed by isoflurane inhalation (Abbott, Wiesbaden Germany), perfused with ice-cold phosphate-buffered saline (pH 7.4, PBS, Sigma-Aldrich, Steinheim, Germany) followed by 4% paraformaldehyde (PFA, Sigma-Aldrich). Brains were post-fixed in 4% PFA overnight. Thereafter, brains were paraffin-embedded and processed into 5μm coronal sections between bregma-0.82mm and bregma -1.82mm according to the mouse atlas.[16] Sections were placed on silane-coated slides, de-paraffinized, re-hydrated and heat-unmasked using citrate buffer, pH 6.0, with 3 times 5 minutes microwave cooking at 600W. Slides were treated with 3% H_2_O_2_ (Roth, Karlsruhe, Germany) diluted in PBS/17% methanol for 30 minutes to block endogenous peroxidase, followed by 1-hour treatment with0.2% Casein (w/v; Sigma-Aldrich) + 0.1% Tween 20 (Sigma-Aldrich) + 0.1%Triton X-100 (Sigma-Aldrich) diluted in PBS for 1-hour. Afterwards, sections were exposed overnight at 4°C to the following primary antibodies diluted in blocking buffer: rabbit polyclonal anti-Iba-1(1:200,Wako, Neuss, Germany), mouse monoclonal anti-synaptophysin (1:50, ab8049, Abcam, Cambridge, UK), rabbit polyclonal anti-Olig-2 (1:1000, ab8109, Abcam), rabbit polyclonal anti-myelin basic protein (MBP)(1:500, ab40390, Abcam), rabbit polyclonal anti-amyloid precursor protein(APP) (1:1000, a8717, Sigma-Aldrich, St. Louis, USA), rat monoclonal anti-glial fibrillary acidic protein (GFAP) (1:1000,13–0300, Invitrogen, Darmstadt, Germany). The next day, sections were incubated with the appropriate secondary antibodies: biotin SP conjugated goat anti-mouse (1:500, ab128976, Abcam), biotin SP conjugated goat anti-rabbit (1:300, Jackson immune research laboratories, Baltimore, USA) or goat anti-rabbit *AlexaFluor*^®^*-488* conjugate (1:500, 1670152, Invitrogen, Rockford, USA) for1hour at room temperature. Slides were developed using the Vectastain elite ABCkit (PK-6104, rat IgG, Vector laboratories, Burlingame CA, USA) applied according to the manufacturer’s recommendations. Diaminobenzidine (DAB, Sigma-Aldrich) was used as chromogen, at 1mg/ml in PBS. Finally, sections were counter stained with hematoxylin for 30 seconds, dehydrated in graded alcohols if required and mounted with Entellan^®^ (Merck, Darmstadt, Germany) or Moviol (Roth, Karlsruhe, Germany).

### Quantification of glial cells and axonal degeneration

Quantification of oligodendrocyte, microglia, astrocyte cell counts, synaptophysin and APP positive bulbs was performed by manually counting the number of positive structures in the medial corpus callosum (CCm). For each animal 2–3 slides were analyzed with a distance of 150μm in between. Cell counts were averaged for each animal and the mean was calculated for each experimental group. All studies were performed at an AxioImager Z2 microscope (Zeiss, Jena, Germany), 20x or 40x lens. Cell numbers are expressed as cells per mm^2^. As an additional parameter for astrogliosis and microgliosis, the surface area covered by GFAP and Iba-1 staining was determined using a densitometric scanning procedure (ImageJ software, Maryland, USA). Results are expressed as relative GFAP orIba-1covered area of the CCm (percentage of the stained area in relation to the non-stained area) as previously described. [17]

### Determination of de-and remyelination

The extent of de- and remyelination in the corpus callosum was examined by staining coronal brain sections of CCm with luxol fast blue (LFB, RAL diagnostics, Martillac, France) and periodic acid-Schiff (PAS) (Merck, Darmstadt, Germany). Sections were graded on a scale from 0 (complete demyelination) to 3 (normal myelin). [18] Anti-myelin basic protein staining was performed as described above to quantify the fluorescence intensity of myelin in the CCm (ImageJ).

### RNA isolation and real-time PCR

The CCm was dissected from whole brains under a light microscope. Total RNA was then extracted from the tissue with Trizol (Life Technologies, Ober-Olm, Germany). First-strand cDNA was synthesized by priming total RNA with hexamer random primers (Life Technologies) and using Superscript III reverse transcriptase (Life Technologies). For quantification of gene transcripts, real-time PCR was performed with SYBR green (Roche Applied Science, Mannheim, Germany) using a 7500 Fast Real-Time PCR System (Life Technologies). The amount of double-stranded PCR product synthesized in each cycle was measured by detecting the FAM dye freed from the SYBR green, which binds to double-stranded DNA. Threshold cycle (Ct) values for each test gene from the replicate PCRs were normalized to the Ct values for *gapdh* control from the same cDNA preparations. Transcription ratios were calculated as 2^(Δct)^ where ΔCt is the difference between Ct (gapdh) and Ct (test gene). Table 1 lists the primers used and the genes analyzed.

**Table 1:** Primer sequences.

Primer	Sequence sense 5' - 3'	Anti-sense 5' - 3'
*gapdh*	ACAACTTTGGCATTGTGGAA	GATGCACGGATGATGTTCTG
*il-1β*	GAAGAAGAGCCCATCCTCTG	TCATCTCGGAGCCTGTAGTG
*FGF-1*	TTTATACGGCTCGCAGACAC	GCTTACAGCTCCCGTTCTTC
*Mrc-1*	TGATTACGAGCAGTGGAAGC	GTTCACCGTAAGCCCAATTT
*FGF-2*	CCAACCGGTACCTTGCTATG	TATGGCCTTCTGTCCAGGTC

### SDS-PAGE and Western blot

Myelin oligodendrocyte glycoprotein (MOG) levels were analyzed using Western blotting. The CCm was dissected from whole brains under a light microscope and bounce-homogenized in a Tris-buffered saline (TBS) solution containing a protease inhibitor cocktail (Roche Applied Science, Mannheim, Germany). Protein concentration was measured using the Bradford assay (Bio-Rad, München, Germany) according to the manufacturer’s protocol. Equal amounts of protein samples were separated by 12% discontinuous sodium dodecyl sulphate-polyacrylamide gel electrophoresis (SDS-PAGE), and transferred onto PVDF membranes (Roche Applied Science, Mannheim, Germany). Membranes were then blocked for 1 hour at room temperature using 10% nonfat dry milk dissolved in PBS. After the 1 hour blocking, PVDF membranes were incubated with mouse anti-MOG (1:2000, MAB5680, Millipore, Darmstadt, Germany) over night at 4°C. Next day, membranes were washed for 30 minutes using Tris-buffered saline containing 0.05% Tween 20 (TBS-T) and were incubated with a peroxidase-conjugated goat anti-mouse (1:3000, P0447, Dako, Hamburg, Germany) secondary antibody for 2 hours at room temperature. Visualization was achieved using the enhanced chemiluminescence method (Western Lightning ECL Substrate, Rodgau, Germany) according to manufacturer’s protocol. For densitometric quantification, the intensities of the specific bands were scanned and normalized to β-actin bands of the corresponding sample using the Image-ProPlus software (Rockville, MD, USA).

### Statistical analysis

Results of each animal were averaged and the mean of each experimental group was calculated. All data are given as means ± SEM. Differences between 2 groups were analyzed by unpaired Student’s t-test. De- and remyelination scores were analyzed by nonparametric Kruskal-Wallis test with Dunn multiple comparisons test and Mann-Whitney test (U-test). Statistical analysis was performed using GraphPadPrism 5 (GraphPad software Inc., CA, USA).Statistical significance was set at p<0.05.At least, 4 animals per group were include for gene expression and protein analysis by realtime-PCR and Western blotting, respectively. Cell and myelin quantifications by immunohistochemistry for cuprizone treated or untreated animals and all different control groups mentioned were performed using at least 5 animals per group. The data presented in this study were obtained from 3 independent experiments.

## Results

### *Asm*-deficiency enhances myelin recovery after acute and chronic demyelination

**Fig 1:**
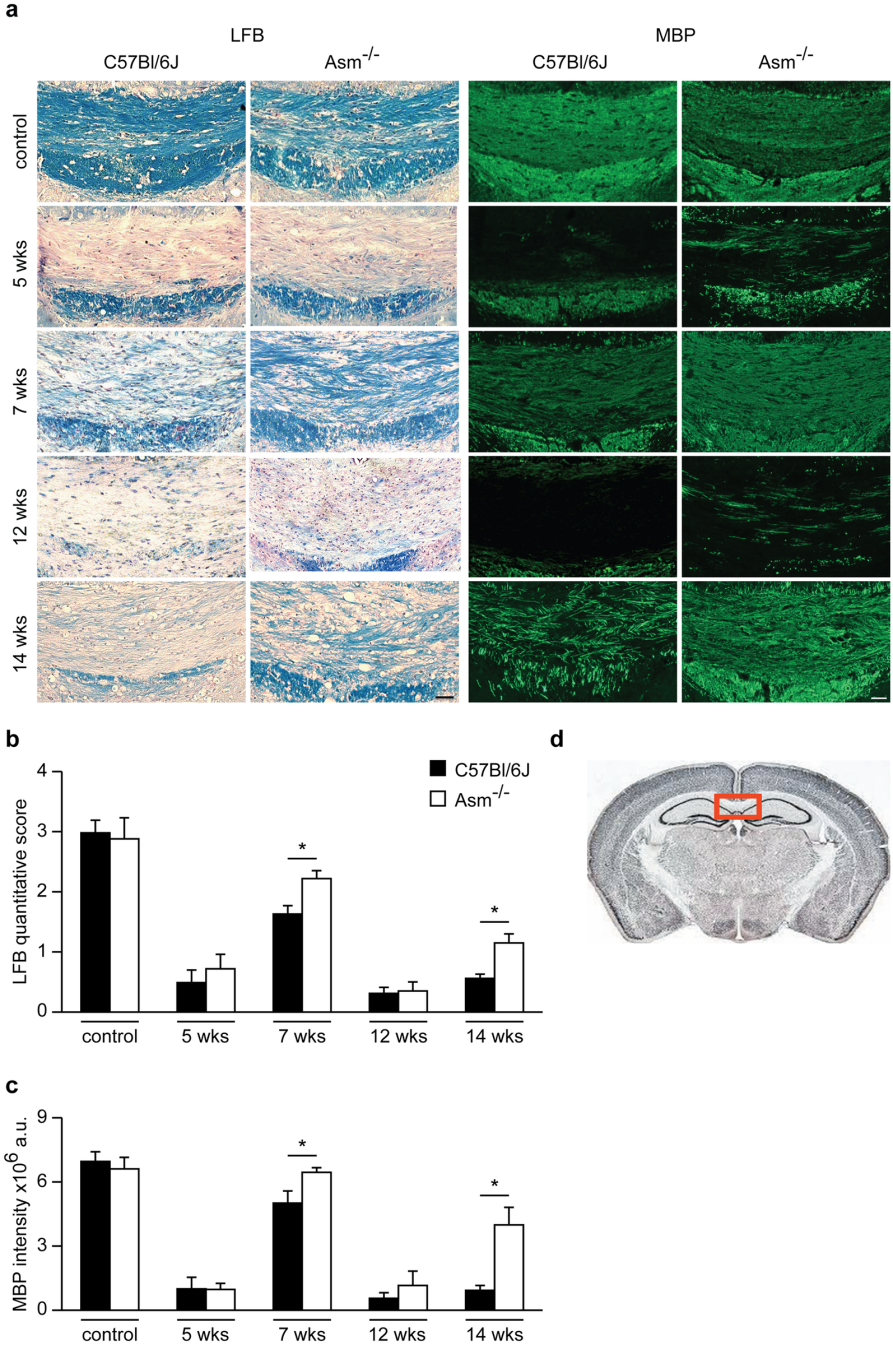
Immunohistochemical staining and quantification of brain sections using LFB and MBP. Cuprizone treatment for 5- or 12-weeks causes a massive decrease in myelination as determined by LFB and MBP staining in the CCm **(a)**. *Asm*-deficient mice showed a significantly increased remyelination compared to wild-type littermates after 2 weeks of recovery following cuprizone treatment as determined by the quantification of myelination score **(b)** and MBP fluorescence intensity in the CCm **(c)**. Representation of the corpus callosum **(d)**.*p<0.05 *Asm*-deficient vs wild-type, scale bar: 100 μm.

**Fig 2:**
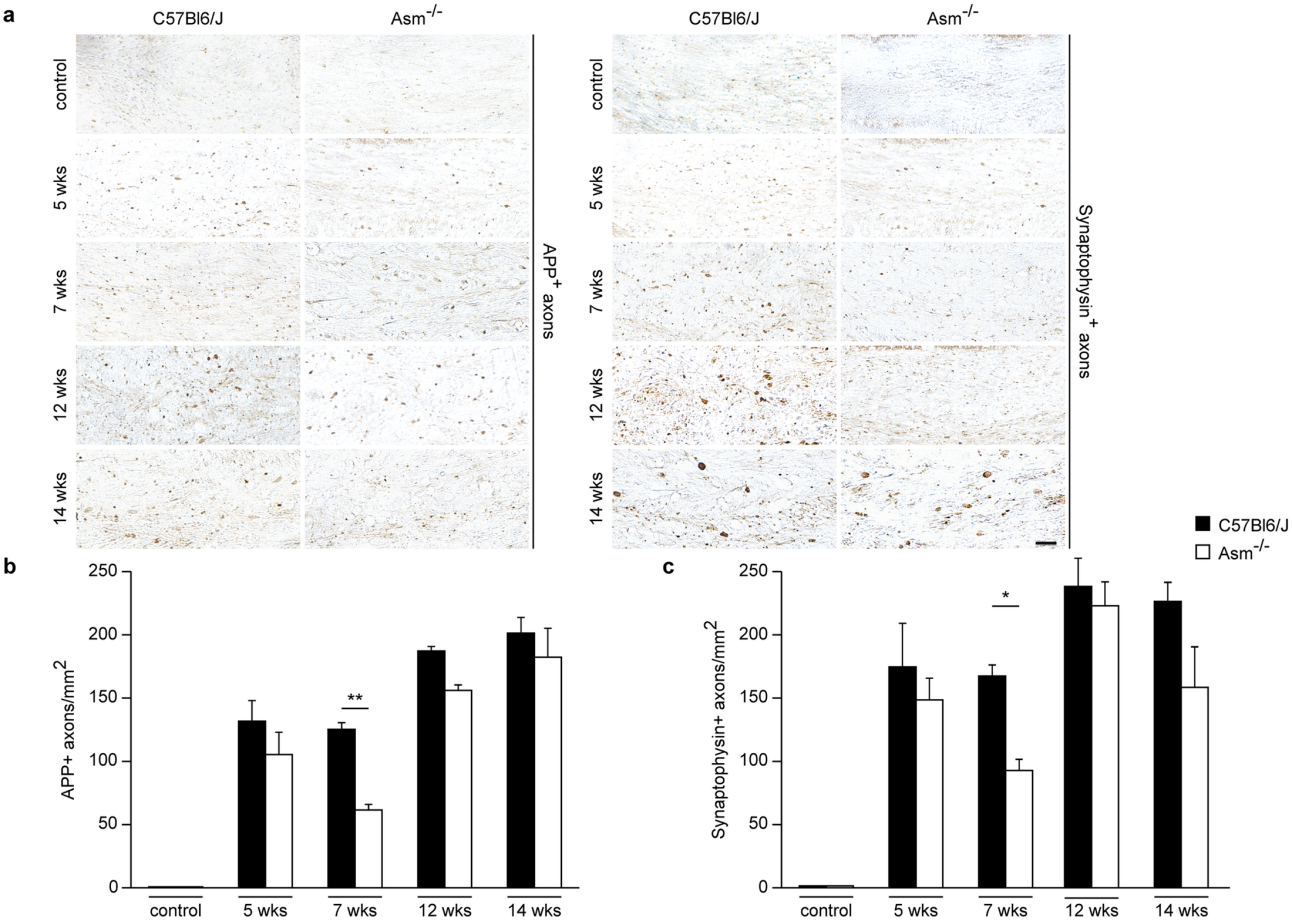
Axonal damage analysis by IHC for APP and synaptophysin. Staining for APP and synaptophysin in the CCm of wild-type and *Asm*-deficient animals after acute or chronic cuprizone treatment followed by 2 weeks recovery period **(a)**. *Asm*-deficient mice showed a significantly lower axonal damage in the 2 weeks recovery phase after acute demyelination (5 weeks cuprizone + 2 weeks recovery) compared to wild-type littermates animals **(b and c)**. However, this difference was not significant after the chronic cuprizone treatment **(b and c)**. ** p<0.01 and *p<0.05 *Asm*-deficient vs wild-types. Scale bar: 100μm.

In order to investigate the role of *Asm* in demyelination, we treated wild-type and *Asm*-deficient littermate mice with cuprizone for 5 weeks (acute demyelination) or 12 weeks (chronic demyelination), followed by 2 weeks of recovery. The extent of demyelination was assessed immunohistochemically by LFB-PAS staining and myelin basic protein (MBP) staining (Fig 1a). Axonal damage was evaluated by APP and synaptophysin analysis (Fig 2a) in the midline of the corpus callosum (CCm), a brain region characteristically affected in the cuprizone mouse model (Fig 1d). Compared to untreated controls, wild-type mice exposedto5- or 12-week cuprizone treatment showed a marked reduction of myelin even after the 2 week recovery period. By contrast, myelin formation in the *Asm*-deficient animals was significantly restored after the 2-week recovery phase, following both 5- or 12-week cuprizone treatment (Fig 1b and 1c).

In animals not given a recovery period, the degree of demyelination observed immediately following 5- or 12- week cuprizone treatment did not differ between wild-type and *Asm*-deficient mice (Fig 1b and 1c).

After acute demyelination induced by 5-week cuprizone treatment, a recovery phase resulted in significant reduction of axonal damage in *Asm*-deficient mice compared to wild-type littermates (Fig 2b and 2c). However, axonal injury was not significantly improved in *Asm*-deficient mice compared to wild-type littermates after chronic demyelination (Fig 2b and 2c).

**Fig 3:**
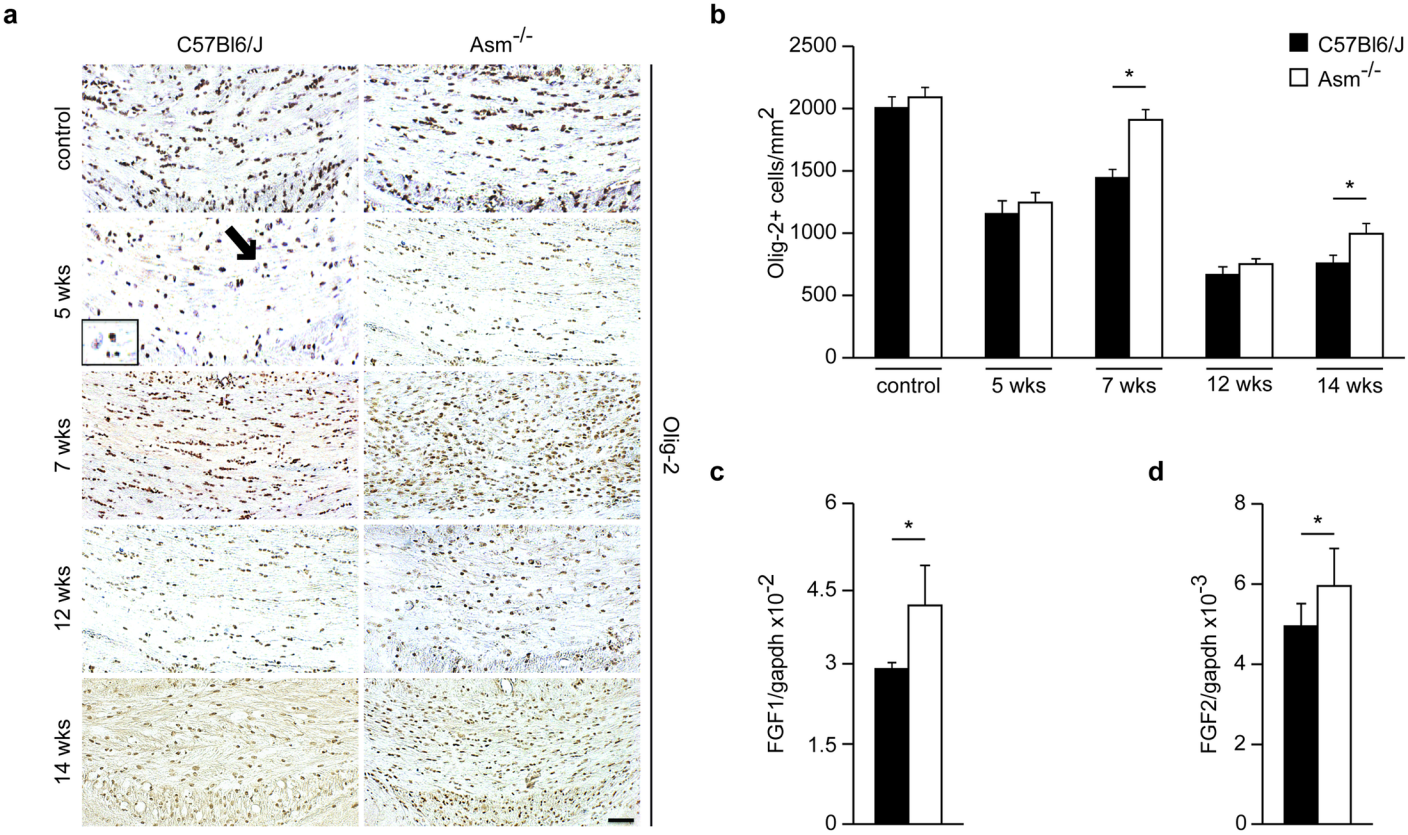
Olig-2 staining for different treatment groups and mRNA analysis of FGF-1 and FGF-2 at week 7. Olig-2^+^ cell numbers were significantly increased in *Asm*-deficient mice compared to wild-type after acute or chronic cuprizone treatment followed by 2 weeks recovery period **(a and b)**. Arrow indicating a strong expression of Olig-2 stain referring to an OPC and nearby a weakly expressed stain by a mature oligodendrocyte. FGF-1 and FGF-2 mRNA levels were also significantly increased after 2 weeks of recovery (week 7) in *Asm*-deficient mice compared to wild-type littermates **(c and d)**. *p<0.05 *Asm*-deficient vs wild-types. Scale bar: 100μm.

Olig-2 is a cellular oligodendrocyte marker, strongly expressed by oligodendrocyte precursor cells (OPCs) and only weakly by mature oligodendrocytes. [19] Compared to wild-type controls, *Asm*-deficient animals showed a significant increase in oligodendrocyte cell numbers in the CCm than wild-type mice after recovery from both, acute and chronic cuprizone treatment (Fig 3a and 3b).

Myelin growth has been shown to be controlled by fibroblast growth factors (FGF). [20, 21, 22] Therefore, FGF-1 and -2 mRNA levels were analyzed. *Asm*-deficient mice showed a significant increase of FGF-and -2 compared to wild-type littermates after the 2 week recovery phase following acute demyelination, hinting towards improved myelin regeneration (Fig 3c and 3d).

### *Asm*-deficiency reduces astrogliosis and production of inflammatory cytokines after acute demyelination

Glial cells, in particular astrocytes, have been discussed to play a dual role in myelin disorders: While they can stabilize synaptic function, they can also promote inflammatory reactions, resulting in myelin and axonal damage. We investigated astroglial and microglial distribution by immunohistochemistry and measured the production of interleukin-1β (IL-1β) and Mrc-1 inflammatory markers by real-time PCR.

**Fig 4:**
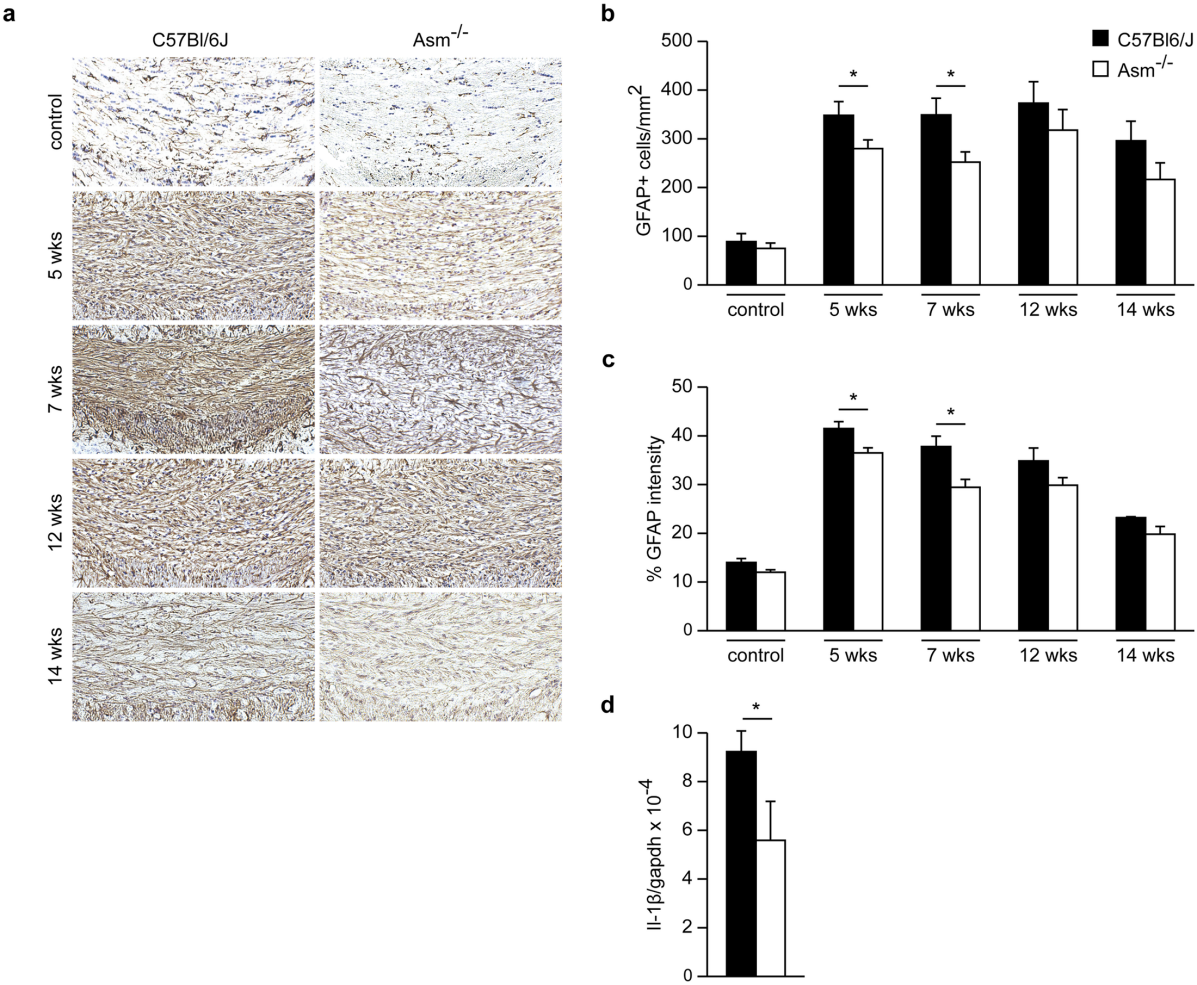
Effect of *Asm*-deficiency on astrogliosis. GFAP stainings in the CCm after 5 or 12 weeks cuprizone treatment followed by 2 weeks remyelination. A significant decrease of astroglial distribution in *Asm*-deficient compared to wild-type littermates after 5 weeks of cuprizone treatment followed by 2 weeks of remyelination was observed **(b and c)**. *Asm*-deficiency significantly decreased Il-1ß mRNA expression levels compared to wild-type littermates after 5 weeks cuprizone treatment followed by 2 weeks recovery **(d)**. *p<0.05 *Asm*-deficient vs wild-type littermates. Scale bar: 100 μm.

GFAP staining intensity revealed a significant reduction in astroglial distribution and astrocytic activation in *Asm*-deficient animals compared to wild-type littermates after the 2-week recovery phase following 5-week cuprizone treatment. This difference was not observed after chronic demyelination (Fig 4a, 4b and 4c). In addition, compared to wild-type littermates, *Asm*-deficient animals showed a significant decrease in pro-inflammatory IL-1β mRNA levels at week 7 (Fig 4d and 4e).

**Fig 5:**
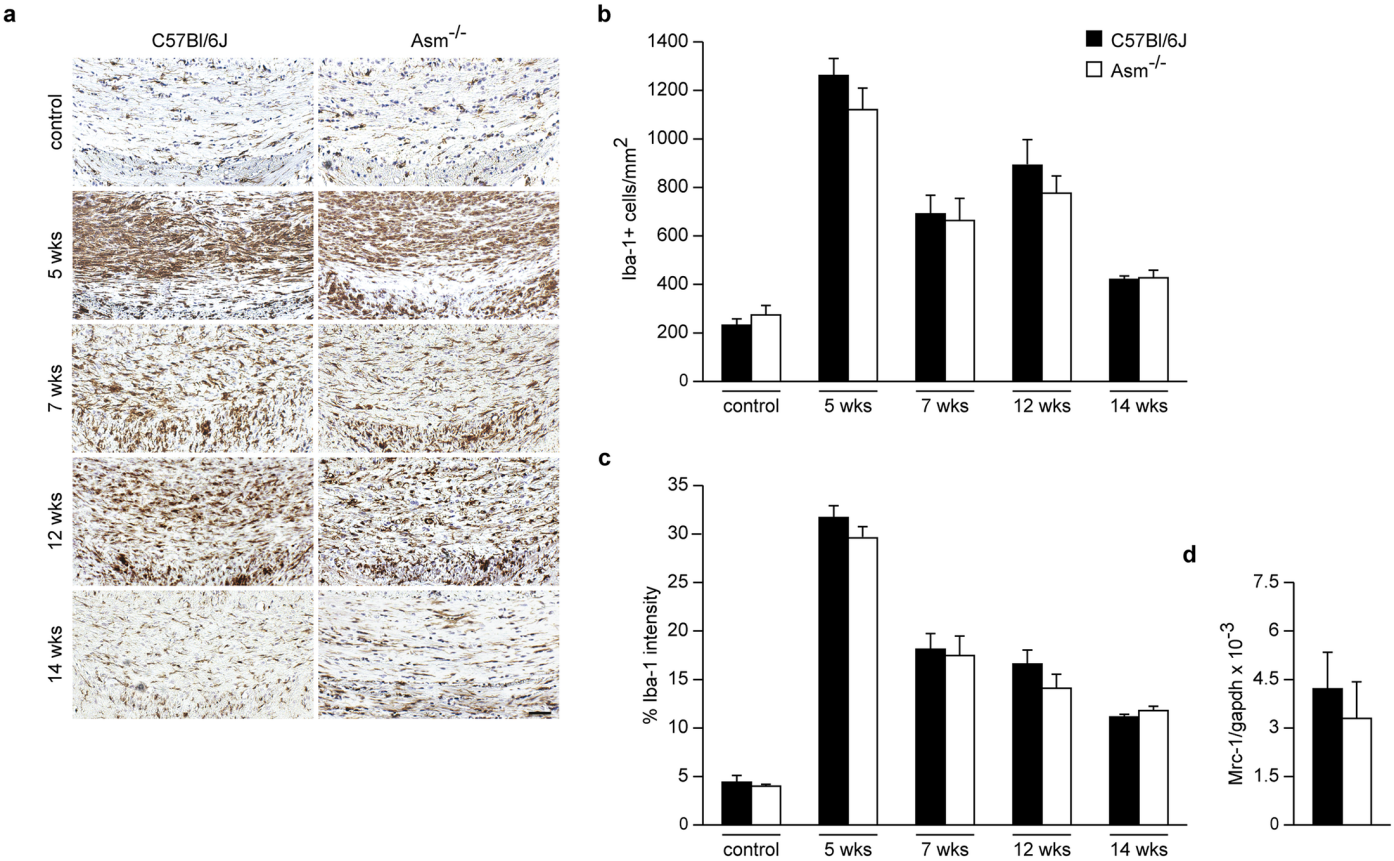
Effect of *Asm*-deficiency on microgliosis and Mrc-1 mRNA levels. Immunohistochemistry for microglia at different time intervals **(a)**. *Asm*-deficiency had no effect on microgliosis neither in acute nor in chronic groups **(b and c)**. mRNA expression levels of Mrc-1 showed no significant difference between the compared groups. Mrc-1 mRNA level expression after 5 weeks demyelination followed by 2 weeks recovery **(d)** *Asm*-deficient vs wild-type littermates. Scale bar: 100 μm.

As shown in Fig 5b, 5c and 5d *Asm*-deficient mice showed no difference in microglial distribution nor in mRNA expression of Mrc-1, a marker correlating toanti-inflammatoryM2 microglia phenotype. [23, 24] Glial distribution in the CCm of untreated healthy control mice was quantified (Figs 4 and 5).

### Pharmacological inhibition of Asm as therapeutic option for myelin repair

**Fig 6:**
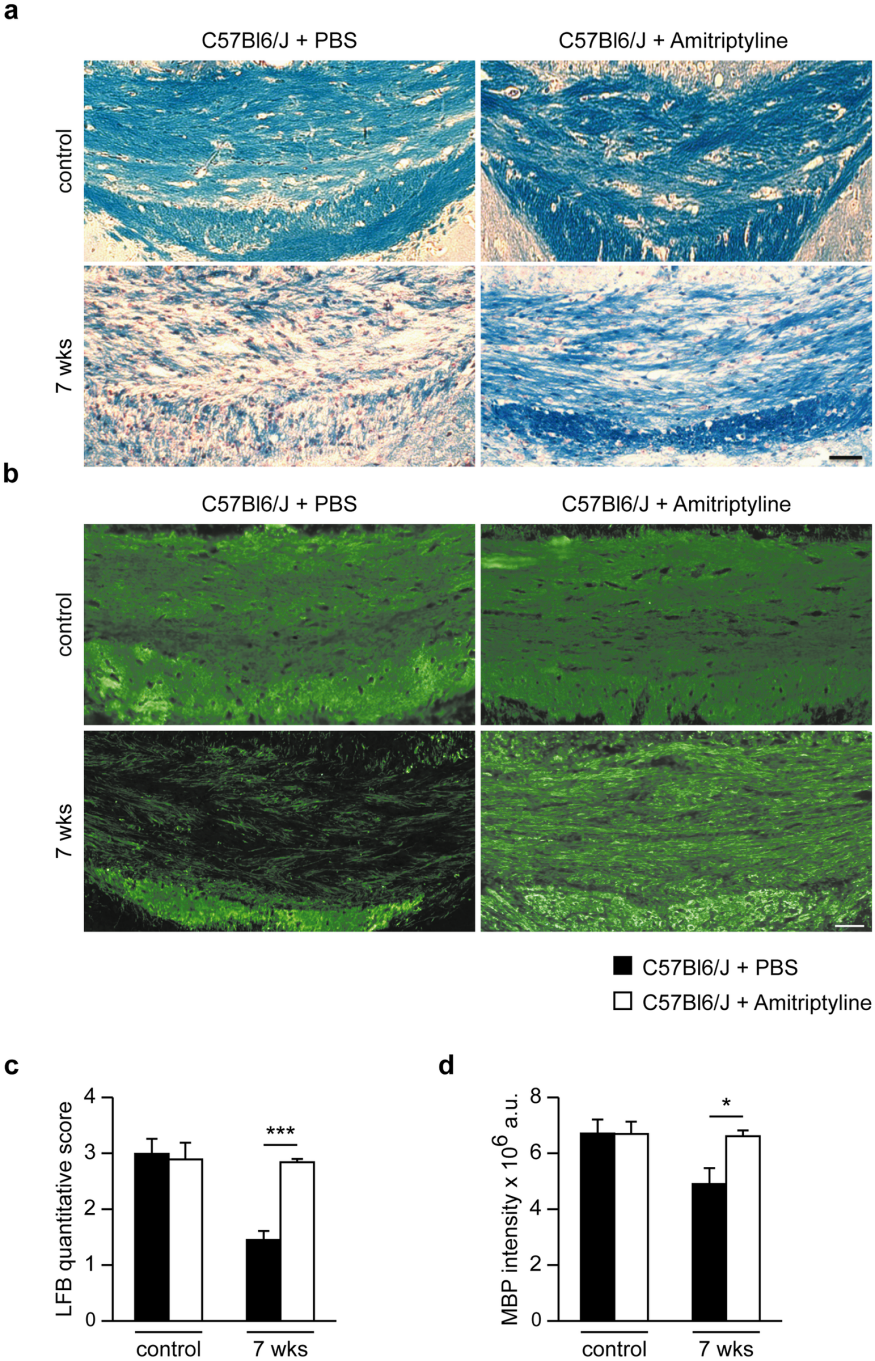
Representative brain sections with CCm stained for LFB and MBP of amitriptyline-treated mice and respective controls. The extent of de- and remyelination was assessed by scoring LFB **(a)** stained brain section and quantifying MBP **(b)** intensity of the CCm. The inhibition of Asm by amitriptyline significantly restored myelin compared to control-treated animals after 5 weeks cuprizone treatment followed by 2 weeks of recovery **(c and d)**. ***p<0.001 and *p<0.05 amitriptyline-treated vs control-treated littermates. Scale bar: 100 μm.

To elaborate a potential therapeutic approach, the effect of pharmacological inhibition of Asm by amitriptyline [25–26] was investigated in wild-type littermate mice in the acute cuprizone model. Amitriptyline injections or PBS (control) injections were administrated twice daily during the 2-week recovery period following 5-week cuprizone treatment. The intensity of LFB and MBP staining suggested a significantly higher myelin recovery in the CCm of mice with amitriptyline-induced Asm inhibition compared to control animals (Fig 6).

**Fig 7:**
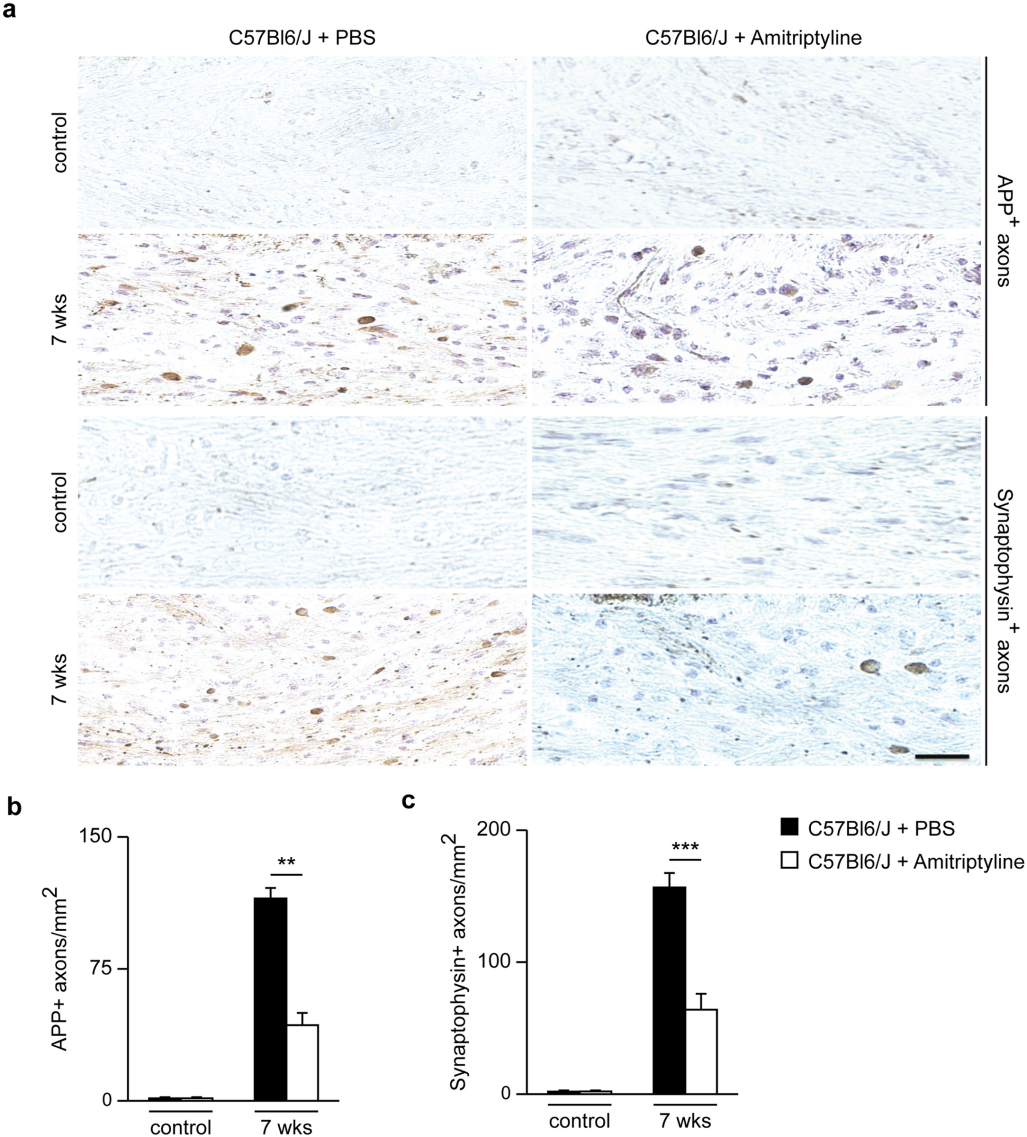
Amyloid precursor protein and synaptophysin detection. Stainings of the CCm of amitriptyline or control-treated animals during the 2 weeks recovery phase after 5 weeks of cuprizone treatment **(a)**. Asm inhibition by amitriptyline showed significantly lower axonal damage, as demonstrated by the quantification of APP and synaptophysin positive buds in the CCm **(b, c)**. **p<0.01 and ***p<0.001 amitriptyline-treated vs control-treated mice. Scale bar, 50 μm.

**Fig 8:**
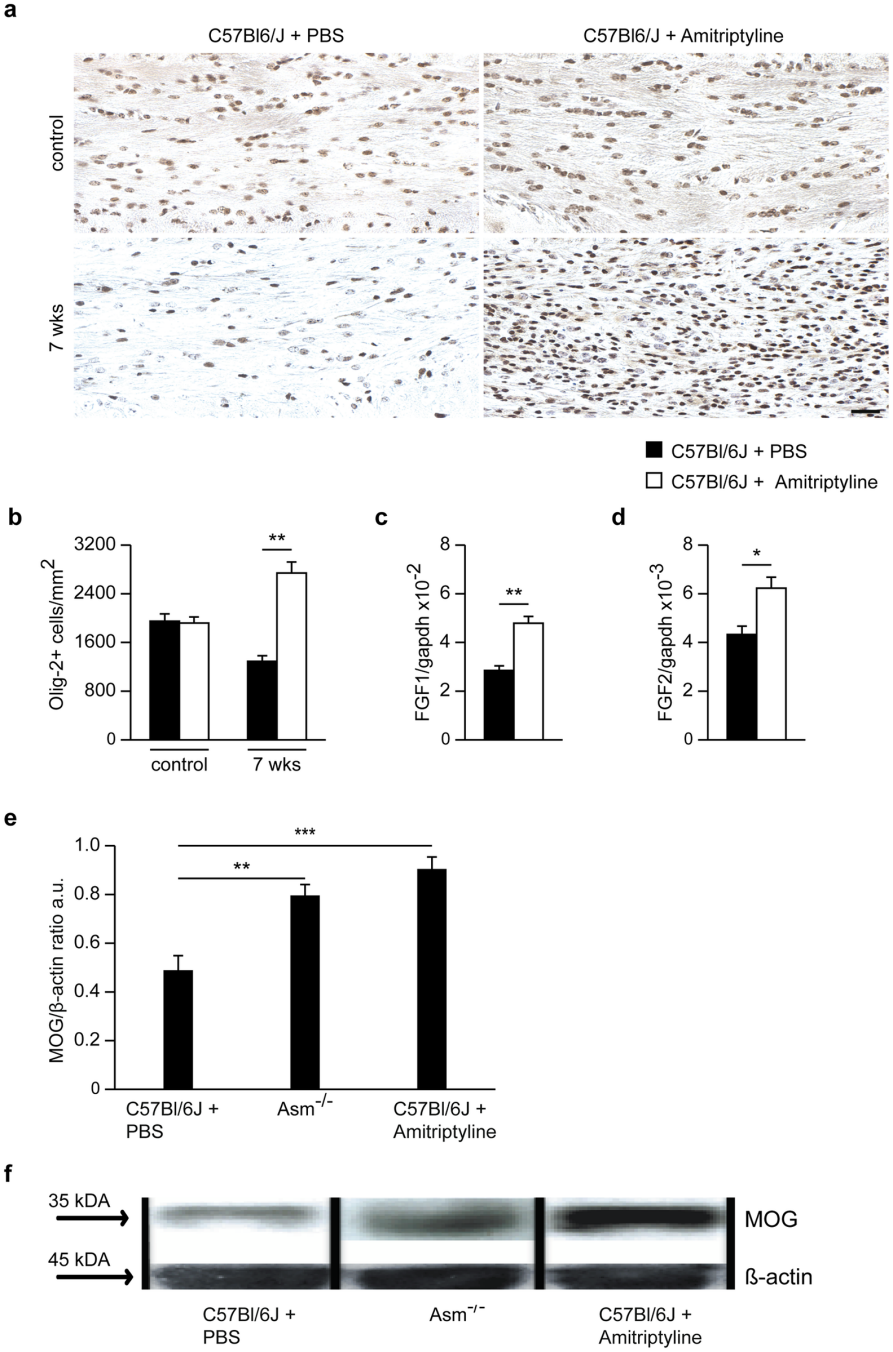
Olig-2 stainings of the CCm of amitriptyline-treated and control treated mice and week 7. Oligodendrocytes in the CCm of amitriptyline-treated mice were significantly increased compared to control-treated animals **(a and b)**. mRNA expression levels of the myelin growth factors FGF1 and FGF2 mRNA levels were significantly increased in amitriptyline-treated mice compared to controls **(c and d)**. Also, protein levels of MOG were significantly higher in amitriptyline-treated mice compared to control-treated littermates at week 7. In addition, *Asm*-deficient mice expressed similar MOG levels as amitriptyline-treated mice **(e and f)**. *p<0.05 and **p<0.01 amitriptyline-treated vs control-treated mice. Scale bar, 50 μm.

Axonal damage in the CCm of animals treated with amitriptyline was significantly lower compared to control-injected mice (Fig 7), as assessed by APP-positive neurons and synaptophysin-positive buds. By contrast, cuprizone-untreated animals did not show any APP- or synaptophysin-positive buds, nor was axonal damage observed (Fig 7b and 7c). After the 2-week recovery period, amitriptyline-treated animals showed higher numbers of olig-2+ cells (Fig 8a and 8b) and higher mRNA levels of the myelin growth factors FGF-1 and -2 (Fig 8c and 8d), compared to control-treated mice. Protein analysis using Western blotting revealed higher MOG levels in the CCm of amitriptyline-injected mice compared to control-treated wild-type animals at week 7. Also, *Asm*-deficient animals after 2 weeks of recovery following 5 weeks of cuprizone treatment showed significantly higher levels of MOG compared to wild-type (Fig 8e and 8f).

**Fig 9:**
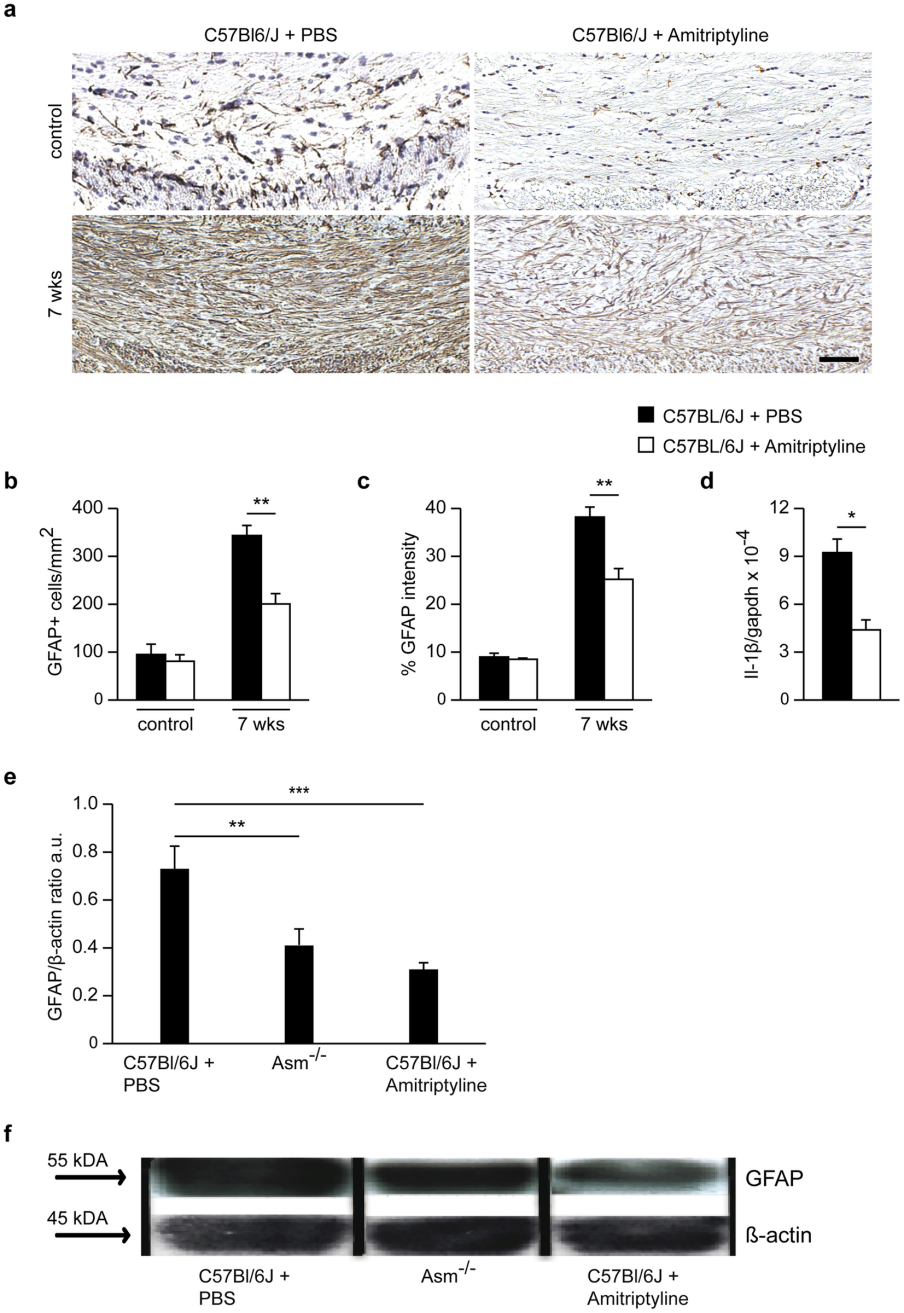
Asm inhibition by amitriptyline for 2 weeks of recovery period after 5 weeks cuprizone feeding effects astrogliosis in the CCm. GFAP^+^ cells and GFAP staining intensity of the CCm significantly decreased in the amitriptyline-treated group compared to control-treated **(b and c)**. Asm inhibition by amitriptyline significantly decreased IL-1ß mRNA expression **(d)**. Western blot analysis revealed that after 2 weeks of recovery with bi-daily amitriptyline-treatment, GFAP protein levels were significantly reduced compared to control-treated littermates. In addition, *Asm*-deficient mice expressed similar GFAP protein levels as amitriptyline-treated mice **(e and f)**.*p<0.05, **p<0.01 and ***p<0.001 amitriptyline-treated vs control-treated groups. Scale bar: 100 μm.

Asm inhibition by amitriptyline also reduced detrimental astrocytosis compared to control animals. Amitriptyline-treated mice showed a significant reduction in astrocyte cell numbers and GFAP staining intensity in the CCm (Fig 9a, 9b and 9c) as well as significantly lower levels of the inflammatory cytokines IL-1β (Fig 9d). Protein analysis at week 7, revealed a significantly lower level of GFAP in amitriptyline-treated mice compared to control-treated littermates. *Asm*-deficient mice after 5 weeks cuprizone treatment followed by 2 weeks recovery showed significantly reduced GFAP levels compared to wild-type animals (Fig 9e and 9f).

**Fig 10:**
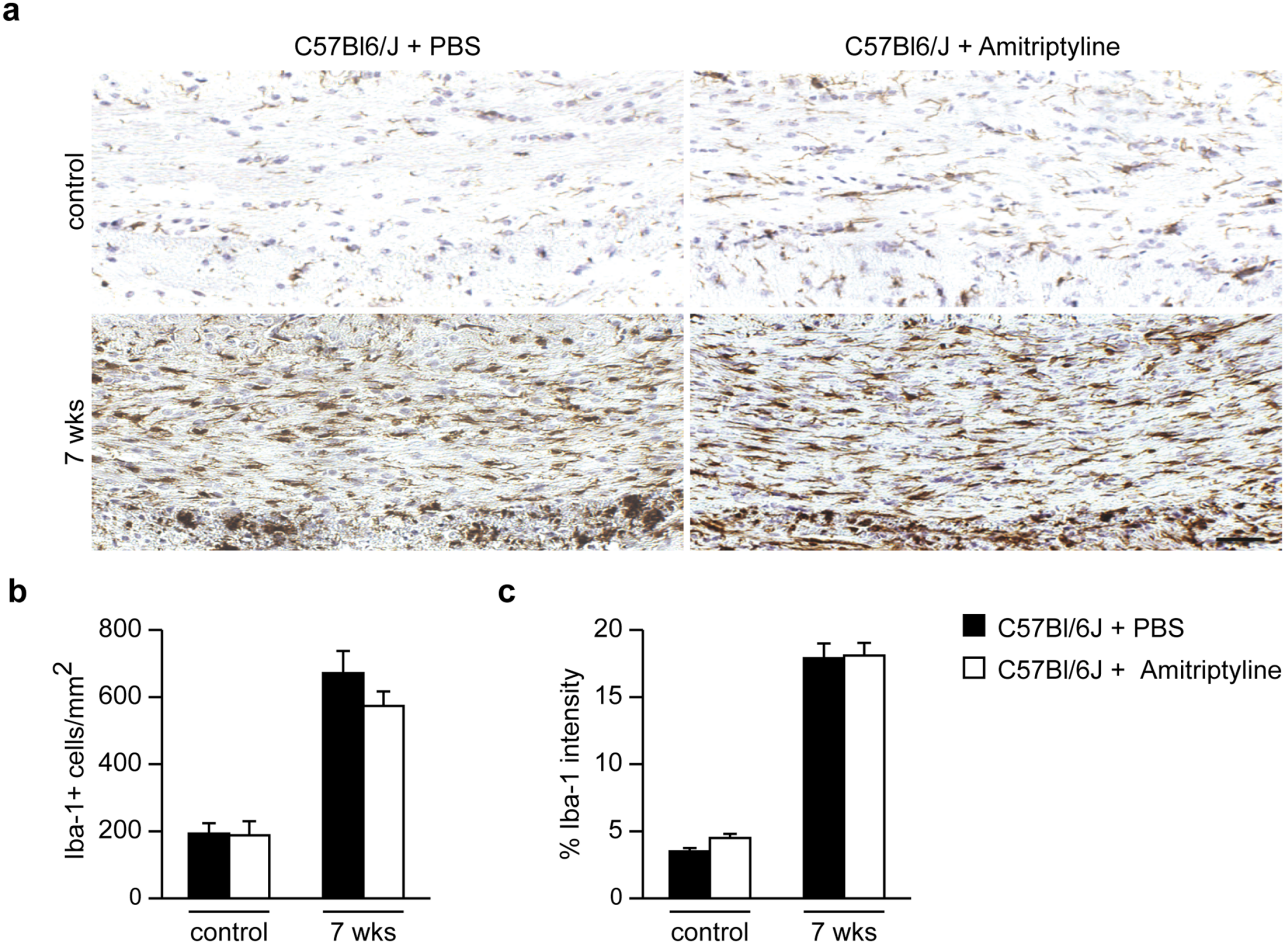
Microglial distribution analysis using Iba-1 staining after Asm inhibition by amitriptyline for 2 weeks of recovery period after 5 weeks cuprizone feeding in the CCm. Iba-1^+^ cell numbers and iba-1 staining intensity in the CCm of amitriptyline- or control-treated animals showed no significance difference **(b, c)**.

However, amitriptyline-treated and control animals showed no significant differences at week 7 in microglial distribution, including cell number quantification and Iba-1 intensity in the the CCm (Fig 10a, 10b and 10c).

## Discussion

Using the cuprizone model, a mouse model used to examine myelin destruction and remyelination in multiple sclerosis, the most common disease of acquired myelin disorders. The results of this study demonstrate that genetic deficiency or pharmacological inhibition of Asm/ceramide system can enhance myelin repair after cuprizone-induced demyelination. In our study, deficiency or blockade of the Asm/ceramide system significantly improved recovery of myelin, as measured by MBP and LFB intensity scores, and increased oligodendrocyte cell numbers, as indicated by increased Olig-2 positive cells in the recovery phase after cuprizone treatment, leading to a reduction of neuronal damage. Asm and its enzymatic product ceramide have been shown to be expressed at higher levels in active human MS lesions compared to unaffected brain tissue. [13, 14]

Interestingly, MBP/LFB levels and Olig-2 positive cell numbers of amitriptyline-treated animals were close to normal values of untreated mice. This finding is in accordance with the observation that ceramide can act together with the pro-inflammatory cytokine tumor necrosis factor alpha to induce oligodendrocyte cell death in human MS brains. [14] In addition, over expression of Asm and subsequent increase of ceramide resulted in reduced neuronal functions even without any further cell stress. [27] Fibroblast growth factors (FGF) are important enhancing factors of myelin growth. [20, 21, 22] Previous studies have shown that FGF blocks Asm activation [28], and the beneficial effects of FGF might be, at least in part, mediated by an interaction with the Asm/ceramide system. Corroborating this hypothesis is our finding that the myelin growth factors FGF-1 and FGF-2 are significantly increased in the recovery phase after acute demyelination in mice lacking *Asm*, suggesting that the Asm/ceramide system negatively regulates FGF after demyelination.

Activation of central nervous system cells, especially astrocytes is hypothesized to be an important mechanism in MS-associated de- and remyelination. [18] Astrocytes can exert a dual role with either beneficial functions (e.g., by facilitating microglial-mediated myelin clearance) [18] or detrimental functions (e.g., by enhancing inflammatory myelin toxic reactions with inflammatory cytokine production). [29]

The significant reduction of activated astrocytes and the decreased levels of IL-1β mRNA in *Asm*-deficient mice suggest a potential role in myelin repair. Interestingly, enhanced levels of Asm mRNA have been described in astrocytes isolated from MS lesions. [13] Although the difference is significant, in our study astrocyte numbers were only 30% reduced in *Asm*-deficient mice compared to wild-type littermates, underscoring the dual beneficial/detrimental role they likely play.

The Asm/ceramide system appears to affect the process of remyelination rather than demyelination. Although a significant difference was detected between *Asm*-deficient and wild-type littermates in GFAP at week 5, the differences observed between *Asm*-deficient and wild-type mice were particularly notable in the recovery phase after acute demyelination (5-week cuprizone treatment), suggesting that the involvement of glial cells is critical primarily after acute myelin injury. In contrast, recovery after chronic demyelination (12 weeks of cuprizone) resulted in an increased myelin content and increased oligodendrocyte cell numbers in *Asm*-deficient mice, but did not show a clear glial cell involvement. These results suggest that other mechanisms are likely involved in chronic demyelination.

Microglia have been reported to play a supportive role in remyelination. [29] In the cuprizone animal model, microglial cells increase in number and become activated after cuprizone treatment. [30] Interestingly, in the recovery phase after acute demyelination, *Asm*-deficient mice and their wild-type littermates showed no difference in microglial distribution and activation, excluding any important role of microglia in myelin repair.

The positive effect of *Asm*-deficiency in myelin recovery can be mimicked by treatment with amitriptyline, a well-known pharmacological Asm inhibitor [25, 26], suggesting a potential therapeutic approach to support remyelination. In our study, amitriptyline treatment was only initiated after cuprizone-induced myelin damage. Amitriptyline treatment resulted in restoration of MBP and LFB intensity to normal values after 2 weeks, with an even higher than normal level of oligodendrocyte cells. These results strongly suggest a detrimental role of the Asm/ceramide system not during myelin damage, but during the repair phase.

## Conclusion

In conclusion, we show that the Asm/ceramide system is strongly involved in remyelination after acute and chronic myelin damage by reducing astroglia distribution and subsequent pro-inflammatory cytokine release. This might lead to the observed enhanced oligodendrocyte proliferation and neuronal preservation.
